# A Novel pH-Tunable Secondary Conformation Containing Mixed Micellar System in Anticancer Treatment

**DOI:** 10.3390/cancers12020503

**Published:** 2020-02-21

**Authors:** Fu-Ying Shih, Wen-Ping Jiang, Xiaojie Lin, Sheng-Chu Kuo, Guan-Jhong Huang, Yu-Chi Hou, Chih-Shiang Chang, Yang Liu, Yi-Ting Chiang

**Affiliations:** 1 Program for Biotech Pharmaceutical Industry, School of Pharmacy, China Medical University, Taichung 404, Taiwan; u106308001@cmu.edu.tw; 2 The Metal Industries Research & Development Centre (MIRDC), Kaohsiung City 811, Taiwan; u101053651@cmu.edu.tw (W.-P.J.); yangliu@mail.mirdc.org.tw (Y.L.); 3 Department of Chinese Pharmaceutical Sciences and Chinese Medicine Resources, China Medical University, Taichung 404, Taiwan; gjhuang@mail.cmu.edu.tw; 4 Department of Chemical Engineering, University of Washington, Seattle, WA 98195, USA; xjlin@uw.edu; 5 Chinese Medicine Research Center, China Medical University, Taichung 404, Taiwan; sckuo@mail.cmu.edu.tw; 6 School of Pharmacy, China Medical University, Taichung 404, Taiwan; houyc@mail.cmu.edu.tw (Y.-C.H.); chihshiang@mail.cmu.edu.tw (C.-S.C.)

**Keywords:** secondary structure, mixed micelle, pH responsive, drug delivery system

## Abstract

In this study, for the first time, we precisely assembled the poly-γ-benzyl-l-glutamate and an amphiphilic copolymer d-α-tocopherol polyethylene glycol succinate into a mixed micellar system for the embedment of the anticancer drug doxorubicin. Importantly, the intracellular drug-releasing behaviors could be controlled by changing the secondary structures of poly-γ-benzyl-l-glutamate via the precise regulation of the buffer’s pH value. Under neutral conditions, the micellar architectures were stabilized by both α-helix secondary structures and the microcrystalline structures. Under acidic conditions (pH 4.0), the interior structures transformed into a coil state with a disordered alignment, inducing the release of the loaded drug. A remarkable cytotoxicity of the Dox-loaded mixed micelles was exhibited toward human lung cancer cells in vitro. The internalizing capability into the cancer cells, as well as the intracellular drug-releasing behaviors, were also identified and observed. The secondary structures containing Dox-loaded mixed micelles had an outstanding antitumor efficacy in human lung cancer A549 cells-bearing nude mice, while little toxicities occurred or interfered with the hepatic or renal functions after the treatments. Thus, these pH-tunable α-helix-containing mixed micelles are innovative and promising for controlled intracellular anticancer drug delivery.

## 1. Introduction

Poly-γ-benzyl-l-glutamate (PBLG), whose structure contains a polypeptide backbone and benzyl side chains, has attracted extensive interest for its biocompatibility and biodegradability [1]. For drug or gene delivery systems, PBLG has commonly been conjugated with hydrophilic polymers into amphiphilic copolymers, where PBLG segments are employed as hydrophobic motifs to stabilize the carriers [2,3]. Notably, the ordered secondary structure driven by the polypeptide backbone is an important feature for PBLG [4]. The α-helix and β-sheet secondary structures are discovered under different conditions. For example, the high molecular weight of PBLG is favored in α-helix structures, while PBLG with a low degree of polymerizations tends towards β-sheet alignment [5]. In fact, these secondary structures are the consequences of the intermolecular or intramolecular hydrogen bond interactions, and thus the conformations of PBLG would be affected by the environmental milieus. It has been reported that the solution polarity strongly influences the helix structures of PBLG [6], and that PBLG showed a helix-coil transformation in different solvents in the presence of an acid, such as trifluoroacetic acid [7,8,9]. Notably, the polymers blended with PBLG have the ability to influence the secondary conformations of PBLG, through either hydrogen bonds or π–π stacking interactions [10,11]. Those performances of the secondary structures would furthermore alter the crystalline alignment of PBLG and play a critical role in the physical properties of PBLG [12,13].

**Scheme 1:**
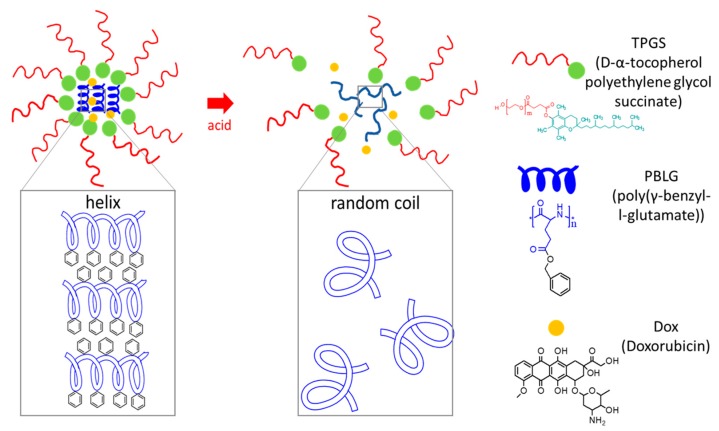
A pH tunable secondary structure containing mixed micelles. The mixed micelles comprising d-α-tocopherol polyethylene glycol 1000 succinate and poly-γ-benzyl-l-glutamate, have interior helix secondary structures due to the polypeptide backbone of PBLG in the neutral conditions. The inner helix structures in the neutral condition enabled to stabilize the micellar structures and encapsulate the anticancer drug doxorubicin. In acidic environment, the secondary structures of mixed micelles would undergo helix-coil transformation to release drug.

As PBLG has a high biosafety and unique secondary structures, for the first time we directly organized the hydrophobic PBLG (M.W. 30–70k Da) with an amphiphilic copolymer d-α-tocopherol polyethylene glycol 1000 succinate (TPGS) into mixed micelles to convey the anticancer drug doxorubicin (Dox). In neutral milieus, such as in the blood or physiological conditions after intravenously administration, the secondary conformation of PBLG within the micellar system is expected to be present, while in mimetic endo/lysosome acidic condition (pH 4.0), the secondary arrangement would undergo transitions into a random coil state (Scheme 1). The interior secondary conformations have been proved by Y. Mochida to stabilize the micellar structures from abrupt disintegration [14]. The micellar density would be regulated as the transitions of the interior secondary structures occurred, representing the potential manner of controlling drug delivery [15], and the interior secondary conformation transitions led to the micelle–vehicle transitions [16]. M. Choi et al. have prepared β-sheet silk nanofilm and controlled the drug liberation via regulating the secondary structure contents, identifying the feasibility of a secondary conformational drug delivery system [17].

This work is the first study that introduces PBLG into an artificially mixed micelle system for controlled intracellular anticancer drug delivery. As we have illustrated previously, the amphiphilic phenolic TPGS and the encapsulated Dox definitely have a predominant impact on both the secondary structures and the crystalline alignment of PBLG within the micellar system. In addition, the terminus of PBLG has a lack of intrahelical hydrogen bonds [8] and is physically incorporated inside the micellar system, thus the response of PBLG toward external pH environments and the releasing profiles needs to be comprehensively investigated. Here, the intracellular drug-releasing and the cytotoxicity toward human cancer cells will also be studied to evaluate the feasibility of these mixed micelles as a novel pH-responsive drug delivery system.

## 2. Results

### 2.1. Preparation and Characterization of pH-Responsive Secondary Structure Contained Mixed Micelles

**Figure 1:**
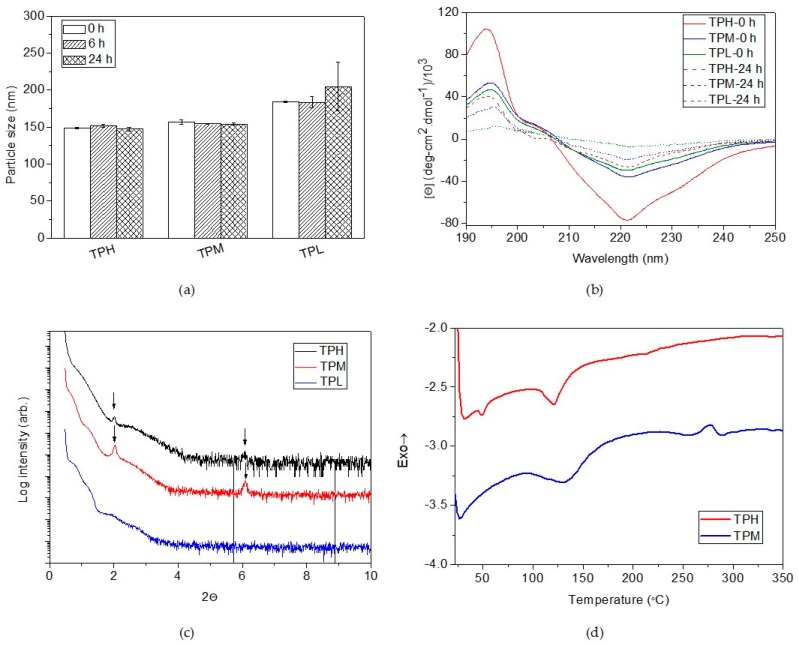
Stability tests of the mixed micelles incubated at 37 °C. (**a**) The changes of particle sizes and distribution at both 6 h and 24 h. (**b**) The CD spectrum of micelles at 24 h for determination of the secondary structures of the mixed micelles. (**c**) The XRD pattern and (**d**) differential scanning calorimetry analysis of micelles for crystalline alignment evaluation.

PBLG and TPGS were weighed at various ratios and dissolved into the N, N-dimethylacetamide (DMAc). The mixed micelles were thereafter prepared via the solvent exchange method. The particle sizes and distributions were measured using dynamic laser scattering (DLS) and the results are shown in Table 1. The critical micellar concentration (CMC) values were also determined using the pyrene probes, also shown in Table 1. The particle sizes of TPL, comprising the highest ratios of the TPGS, were 184.0 ± 0.7 nm, and the particle sizes of the mixed micelles which had the lowest ratios of the TPGS (TPH) were 148.7 ± 1.3 nm. The mixed micelles whose particle sizes were 157.0 ± 3.0 nm presented equal weight ratios of the PBLG and TPGS. All the micelles exhibited low polydispersity (PDI) value, representing the monodispersity of these micelles. TPH micelles exhibited not only the smallest particle size but also the lowest CMC value (5.68x10^−4^ mg/mL). The CMC value of TPM (3.22x10^−3^ mg/mL) was slightly higher than that of TPL mixed micelles (1.29x10^−3^ mg/mL). The CMC value was considered as the majority in the stability of the mixed micelles [18]. Therefore, the stability of these mixed micelles at 37 °C was evaluated through the hydrodynamic diameter changes, and the results are shown in Figure 1a. TPL micelles, whose CMC was higher than TPH mixed micelles, exhibited relatively significant particle size changes, and the particle size increased until around 205 nm, after incubation at 37 °C for 24 h. The hydrodynamic diameters of TPM and TPH did not show obvious particle size changes, indicating that the TPM and TPH mixed micelles showed a high stability at 37 °C. It is worth noting that the CMC value of the TPM micelle was five times higher than that of the TPH micelle and even also higher than TPL mixed micelles, while the hydrodynamic diameters did not significantly increase upon incubation. PBLG was previously reported as folding into specific secondary structures and the inner secondary structures of the micelles have been identified related to the particle stability [14,19]. Since the stability was more connected with the PBLG contents, instead of the CMC value, the role of PBLG in the mixed micellar system was investigated in our study.

For the hierarchical conformation analysis of PBLG in these mixed micelles, circular dichroism (CD) spectroscopy was further applied from the wavelength of 190 to 250 nm to identify the PBLG participation and its secondary folding. The CD spectra of these mixed micelles with or without incubation at 37 °C for 24 h is shown in Figure 1b. Before incubation at 37 °C, the mixed micelles all exhibited a negative band around 222 nm, as well as a positive band at approximately 195 nm. The valley around 222 nm represented the α-helix conformation. However, another characteristic negative peak for α-helix conformation was not observed at 208 nm in the CD spectrum because the randomly coiled polypeptides also existed [9]. The single minimum spectrum was also observed in all groups, while the magnitudes of the 2 bands within the mixed micelles were dependent upon the TPGS and PBLG ratios. The α-helix conformation contents increased with the increasing PBLG ratios within the mixed micelles, evidencing the PBLG folding inside the micelles with helical folding, whereas the increasing TPGS ratios would lead to the random coil state within the inner micellar structures. TPH exhibited the highest α-helix content among all the micelles, due to the strong magnitude of the valley at 222 nm. The mixed micelles were further incubated at 37 °C for 24 h. The CD spectrum, also shown in Figure 1b, revealed the weakened magnitude of the negative peak at 222 nm because of the lowering of the α-helix conformational contents. In particular, for TPL, the random-coiled state was almost dominant, whereas the helical domains still could be detectable in TPH and TPM mixed micelles. The results clearly point out that the secondary folding may have the main role in stability.

Based on the results of the particle sizes and secondary structure featuring in the CD spectrum, we focused on TPH and TPM mixed micelles to investigate their inner microstructure. The mixed micellar structures were mainly identified with differential scanning calorimetry (DSC) thermograms after mixed micelles were incubated at 37 °C for 24 h. In the TPGS thermograms in Figure S1 in the Supporting Information, two endothermal peaks at 40 and 317 °C can be observed, representing, respectively, the melting point (T_m_) and the decomposition temperature. The PBLG thermogram shows one sharp endothermal peak at 311 °C, indicating its T_m_. However, for TPH and TPM mixed micelles, after incubation at 37 °C for 24 h, the endothermal peaks at 317 and 311 °C were undetectable, which could be attributed to the micelle formation. These results show that the secondary structures within the micellar structures play a crucial role in micellar stability.

Despite TPH and TPM mixed micelles having adequate stability in the mimetic physiological environment, they exhibited subtle distinctions after incubation at 37 °C for 24 h according to the transmission electron microscopy (TEM) images with phosphotungstic acid (PTA) staining (Figure S2 in the Supporting Information). The core–shell structures could be observed in both TPH and TPM mixed micelles. However, TPM mixed micelles were observed with relatively loose structures, due probably to the lower α-helical conformation contents and higher CMC value (Figure 1b). The regular secondary structures may lead to the formation of liquid crystalline structures [20]; crystalline structures have been reported in the microphase of PBLG [21] and they would affect the strength and stability of the micelles [22]. Thus, the crystalline structure induced by helix conformation within the TPH and TPM mixed micelles was investigated in our study. The crystalline structure within the mixed micelles was analyzed using an X-ray diffractometer (XRD) and DSC. The XRD pattern of TPH and TPM mixed micelles in Figure 1c showed 2 peaks at 2θ = 2 and 6, while no peak was detectable for TPL mixed micelles. The results could be reasonably explained by the secondary architectures. The ordered α-helix structures in TPH and TPM mixed micelles enabled the formation of the inner crystalline structure and led to a higher stability. The distinctions of the crystalline microphase between TPH and TPM mixed micelles from TEM images after incubation at 37 °C for 24 h were further identified via the DSC thermograms in Figure 1d. TPH mixed micelles exhibited 2 sharp endothermal peaks around 50 and 120 °C that indicate the melting temperature, respectively, demonstrating the highly crystalline structure. The TPM thermogram showed one broad endothermal peak around 132 °C and a sharp endothermal peak at 277 °C, demonstrating the interior semicrystalline structure within TPM mixed micelles. TPH mixed micelles possessed high crystalline alignments, even after being incubated at 37 °C, indicating that micellar structures still could be maintained; although the secondary structure of TPM mixed micelles still could be detected, only the semicrystalline structure was aligned within the TPM mixed micelles, inducing the looser structures after 37 °C incubation. The secondary structure could regulate the interior crystalline behaviors of the mixed micelles and further affect the micellar stability.

### 2.2. pH Responsiveness of the pH-Responsive Secondary Structure Contained Mixed Micelles

**Figure 2:**
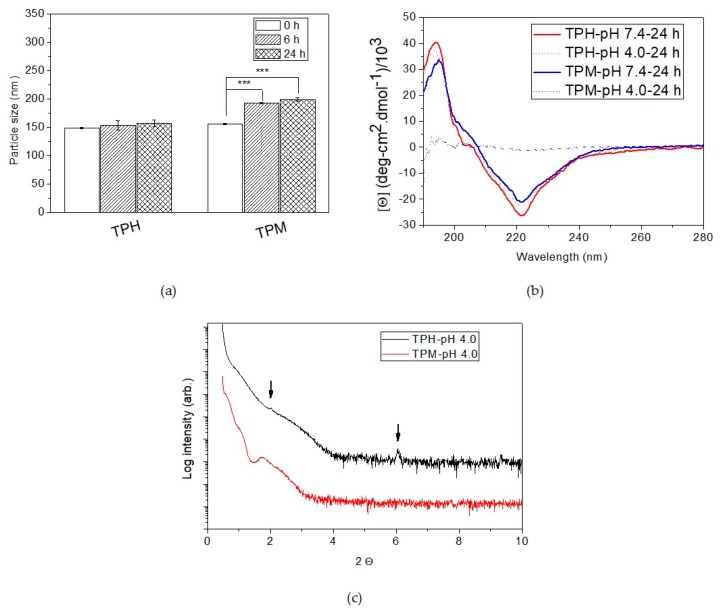

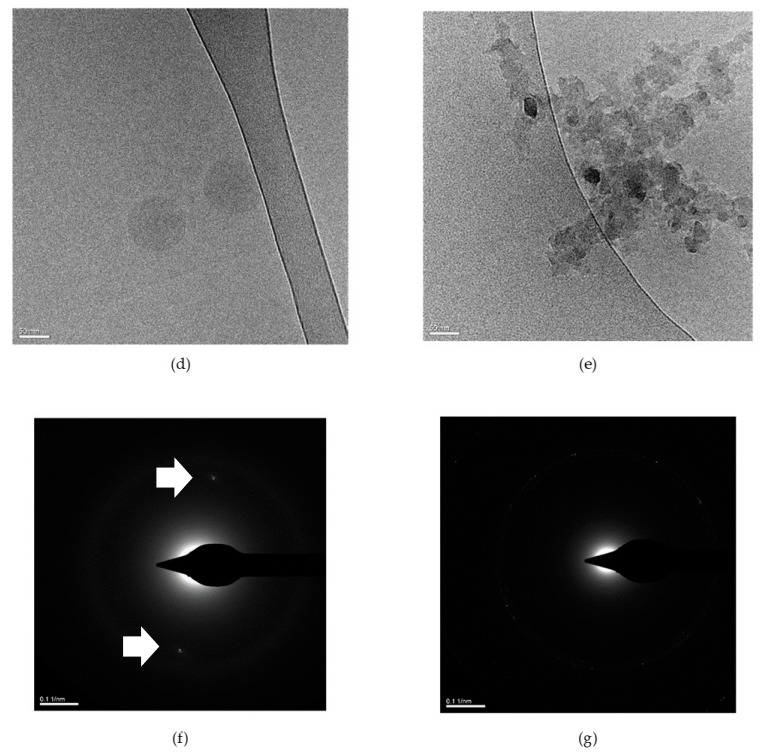
pH-Responsiveness of the mixed micelles. (**a**) The particle sizes of the TPH and TPM mixed micelles were measured by DLS in pH 4.0 condition after incubation at 37 °C for 6 and 24 h. (**b**) The CD spectrum of micelles were conducted to determine their secondary structures. (**c**) XRD patterns of the TPH and TPM mixed micelles after acidic treatment for 24 h. A cryoTEM was utilized for morphological observation of TPM micelles after incubation at pH 7.4 (**d**) and 4.0 (**e**) for 24 h. The diffraction images of TPM mixed micelles at pH 7.4 (**f**) and pH 4.0 (**g**) were simultaneous undergone to further conduct the crystalline alignment. Asterisk markers indicate significant difference in statistics (***p < 0.005).

The secondary structure of PBLG was reported as having a responsive ability toward the protons in solutions. [23]. The pH-responsive behaviors of PBLG could be assumed by tuning the secondary structures. Since TPH and TPM mixed micelles exhibited little particle size change and high stability, the pH responsiveness was investigated to evaluate the potential for a pH responsive drug delivery system. First, the hydrodynamic diameters of TPH and TPM mixed micelles at pH 4.0 condition in 37 °C were examined using DLS, as shown in Figure 2a. TPM mixed micelles increased in particle size upon incubation. After 6 h incubation, the particle sizes enlarged from around 155 nm to 193.33 ± 1.10 nm, and the particle sizes kept increasing to 199.30 ± 2.93 nm at 24 h post-incubation. Conversely, TPH micelles did not exhibit significant particle size changes, even after being incubated for 24 h. The CD spectrum shown in Figure 2b revealed that the helix conformation of TPH still remained after 24 h of acidic treatment, while the α-helix structures were undetectable for the TPM micelles. TPM mixed micelles were also incubated at pH 6.5 condition, which was imitate the tumor tissue environment, and the CD spectrum were also investigated, shown in Figure S3 in the Supporting Information. The valley at 222 nm of TPM mixed micelles incubated at pH 6.5 was intermediate between that of mixed micelles incubated at pH 7.4 and pH 4.0, while the valley was closer than the position of mixed micelles incubated at pH7.4. The results further identified TPM mixed micelles were sensitive to outer milieus but in mimetic tumor environment (pH 6.5), TPM mixed micelles possessed similar inner structures to those in pH 7.4 condition. The XRD pattern was also examined in Figure 2c. The peaks at 2θ = 2 and 6 were canceled in the TPM micelles, while they were still detectable in the TPH micelles. The DSC thermograms were also evaluated, as shown in Figure S4 in the Supporting Information. For TPM mixed micelles, the combinational endothermal peaks from 310 to 323 °C were detected, resulting from the TPGS decomposition and the Tm of PBLG. This evidenced the loss of interactions between polymers within micelles and the crumbling of the TPM mixed micelles; for TPH mixed micelles, only one peak at 297 °C was observed, possibly showing that only fractional crystalline domains were disordered.

The crumbling of the TPM mixed micelles was further directly observed using a cryoTEM. The images are shown in Figure 2d,e. Figure 2d shows the TPM mixed micelles without acid treatment and the complete micelles can be observed, demonstrating that TPM micelles after incubation at 37 °C in pH 7.4 for 24 h can still maintain their architectures. The cryoTEM images in Figure 2e indicate that TPM micelles collapse after acidic treatment. In addition, the XRD pattern images, as shown in Figure 2f, also indicate that, after incubation at the neutral condition for 24 h, the spot-like crystalline diffractions could be discovered within the TPM mixed micelles. The diffraction pattern of the TPM mixed micelles is represented in the ordered crystalline structure, aligning with the out-of-plane direction [24]. The crystalline spots disappeared in the XRD pattern image of the acid-treated TPM micelles in Figure 2g, demonstrating that the crystalline nanodomains in TPM mixed micelles would be disordered at pH 4.0, in harmony with the results of the XRD pattern in Figure 2c. The gel permeation chromatogram (GPC) in Figure S5 in the Supporting Information shows that the molecular weight of the mixed micelles did not change after acidic treatment for 24 h, demonstrating that neither TPGS nor PBLG in the mixed micelles were degraded by acids. The disordered alignment of PBLG and the inducing micellar deformation were the main cause for the pH responsiveness of the mixed micelles, whereas the pH sensitivity of PBLG, however, was mostly neglected in other polymeric micellar systems [1,23,25]. The pH-tunable secondary structure containing mixed micelles was further studied in application as a novel drug delivery system.

### 2.3. Doxorubicin-Loaded Secondary Conformation Contained Mixed Micelles Preparation and Drug-Releasing Behaviors

**Figure 3:**
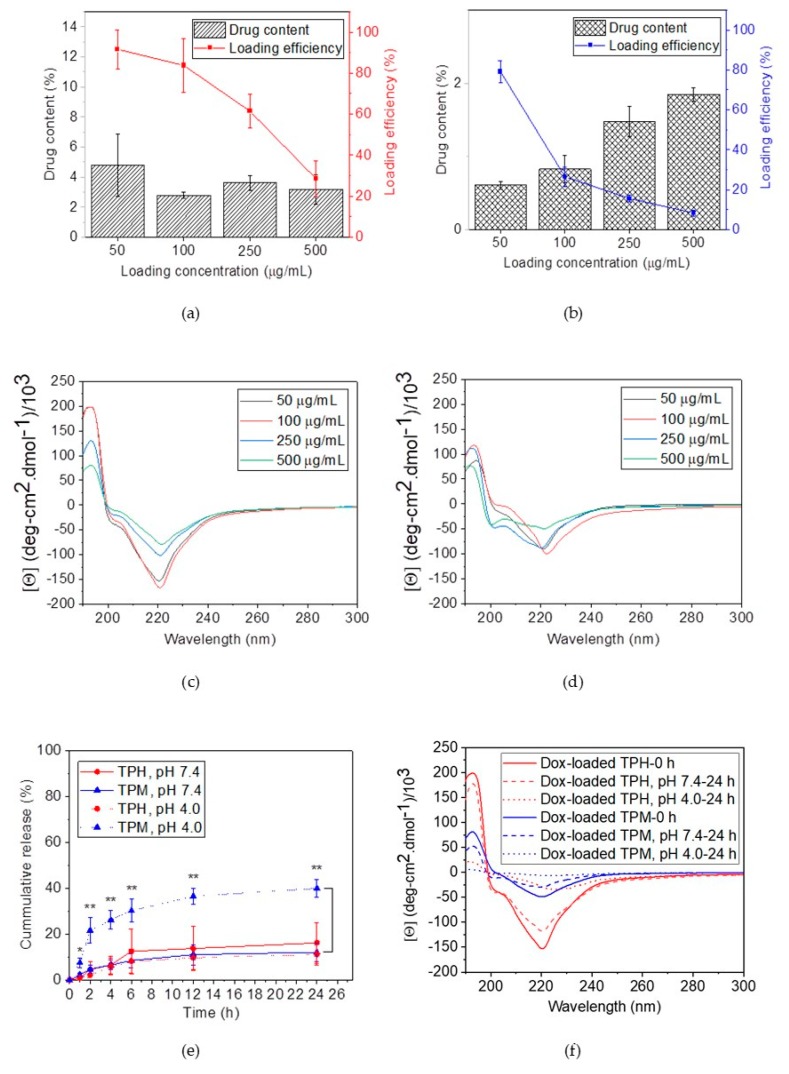
Drug loading and releasing behaviors. The anticancer drug doxorubicin (Dox) was encapsulated into TPH and TPM polymeric mixed micelles. To optimize the drug loading efficiency, various amounts of the doxorubicin were in-feed and the drug contents and efficiency of TPH (**a**) and TPM mixed micelles (**b**) were determined by a UV-vis spectrum. Simultaneously, CD spectrum of TPH (**c**) and TPM (**d**) micelles were also detected. The drug releasing profiles of these mixed micelles at 37 °C in pH 7.4 and 4.0 conditions were also determined using a UV-vis spectrum (**e**). The CD spectrum of the mixed micelles after incubation at pH 7.4 and 4.0 conditions for 24 h were also investigated (**f**). Asterisk markers represent significant difference in statistics (**p < 0.01).

TPM mixed micelles have been identified as stable at 37 °C and pH responsive at pH 4.0, qualities which are considered to be underlying principles for a drug delivery system in anticancer therapy. TPH mixed micelles exhibited high stability at pH 7.4 condition, also indicating potential as a drug delivery system. Both TPH and TPM mixed micelles were herein chosen to encapsulate the anticancer drug doxorubicin (Dox) for further study. The anticancer drug doxorubicin hydrochloride was first reacted with triethylamine (TEA) in DMAc and then dissolved together with PBLG and TPGS, forming the doxorubicin-loaded mixed micelles (Dox-loaded mixed micelles) through the solvent exchange procedure. In order to optimize the loading efficiency and drug contents in the mixed micelles, the various doxorubicin concentrations (50, 100, 250, and 500 μg/mL) were fed into the polymer–DMAc mixtures for mixed micelles preparation, and the loading doxorubicin was determined using an ultraviolet–visible light spectrometer (UV-vis spectrometer). The drug contents and loading efficiencies of TPH and TPM mixed micelles are respectively shown in Figure 3a,b. The drug contents in TPH mixed micelles remained steadily around 3%, even as the increasing levels of Dox were employed, while their loading efficiencies decreased along with the loading concentration. The maximum loading efficiency was 91.5% when 50 μg/mL of Dox was added. For TPM mixed micelles, the maximum drug loading efficiency was approximately 80% when 50 μg/mL of Dox was applied, while the drug contents were 0.5%. The maximum drug content of TPM mixed micelles was almost 2% when 250 μg/mL of Dox was introduced, while the loading efficiency reduced to less than 10%.

The CD spectrum also indicates that the secondary conformations within the TPH and TPM mixed micelles changed with the loading concentration of Dox in Figure 3c,d, respectively. With respect to the TPH mixed micelles in Figure 3c, the helix–coil hybrids were detected in various Dox loading concentrations. However, the helical contents, determined by the valley at 221 nm, decrease along with the loading concentration. The CD spectrum of TPM mixed micelles in Figure 3d exhibits distinguishing characteristic peaks upon the addition of Dox loading solutions. Figure 3d shows the results of when 50 μg/mL of Dox infeed solution was treated with the mixed micelles of an α-helix conformation, whose valley was detected at 221 nm. As the loading Dox concentration increased to 100 μg/mL, a helix–coil conformation still could be determined, while the negative peak slightly moved to 222 nm. As the Dox concentration further increased, toward 250 and 500 μg/mL, the mixed micelle exhibited two negative peaks at 200 and 220 nm and one positive peak at 192 nm, indicating the fairly large number of random-coil structures [9]. Considering the loading efficiency as well as the secondary structures of Dox-loaded mixed micelles, eventually, we chose the 50 μg/mL of Dox in the feed concentration to fabricate the Dox-loaded TPH and TPM mixed micelles to further study their drug-releasing profiles.

Figure 3e presents the drug-releasing profiles of Dox-loaded TPH and TPM micelles at 37 °C in pH 7.4 and 4.0 conditions. At pH 7.4, both the mixed micelles released low levels of Dox. After being incubated at pH 7.4 for 24 h, only approximately 10% of Dox was liberated from TPM mixed micelles, and 15% of Dox was released from TPH mixed micelles. Meanwhile, Dox-loaded TPM mixed micelles at pH 4.0 exhibited an abrupt releasing behavior within the first two hours and a continuous releasing curve until 24 h post-incubation. A total of 40% of the Dox was released from the TPM mixed micelle in acidic conditions. Regarding the TPH mixed micelles, the drug-releasing behavior at pH 4.0 condition was nearly close to that at pH 7.4 condition, demonstrating the lack of pH responsiveness of TPH mixed micelles. The drug-releasing behaviors were considered relevant to the secondary conformation. The CD spectrum of Dox-loaded TPM mixed micelles in Figure 3f shows that at pH 7.4 for 24 h, the α-helix conformation remained dominant. After acidic treatment for 24 h, the proportions of the random coil state increased. The CD spectrum of Dox-loaded TPH mixed micelles also exhibited the pH responsive alternation in magnitude. However, after acidic treatment for 24 h, the helical domains of TPH mixed micelles remained more than those of TPH mixed micelles. The result identified that the alteration of secondary conformation within TPM mixed micelles at various pH conditions could lead to facilitating drug releasing, whereas the remaining secondary structures of TPH mixed micelles halt the rapid drug release at acidic condition.

### 2.4. In Vitro Tests

**Figure 4:**
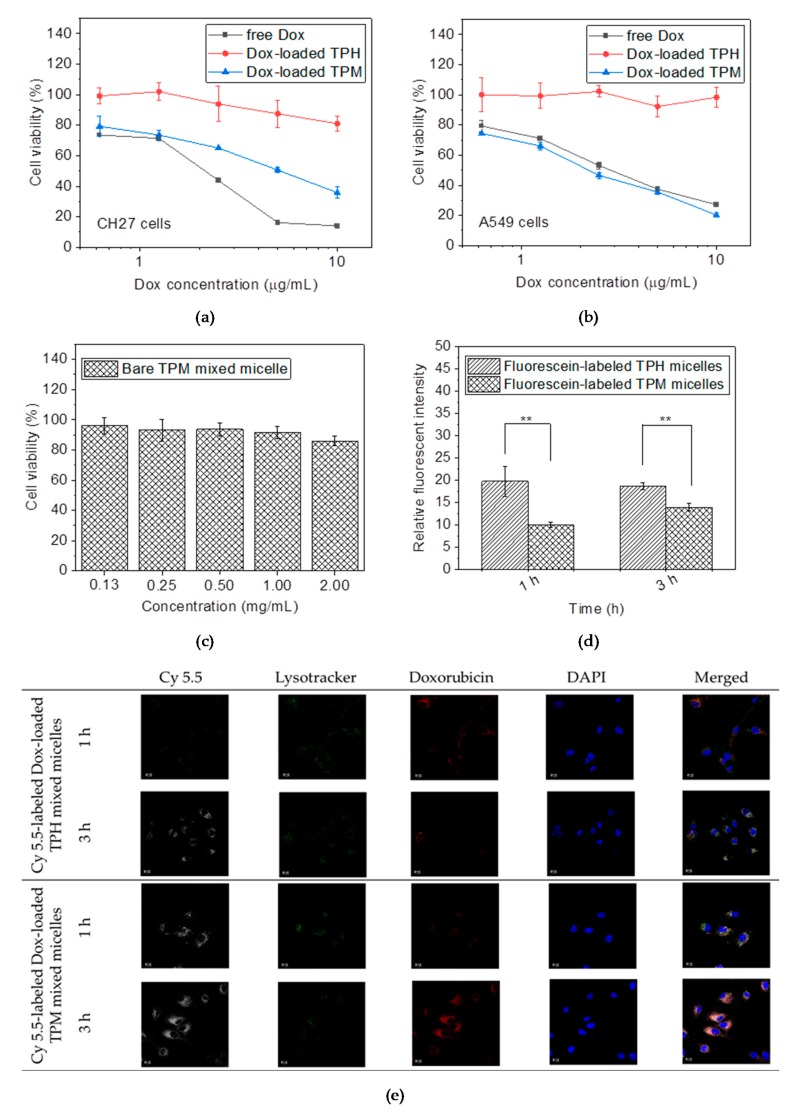
In vitro tests for Dox-loaded micelles contained secondary structures. Inhibitory effects of the free Dox, Dox-loaded TPM and Dox-loaded TPH micelles after incubation with human lung cancer CH27 (**a**) and A549 cells (**b**) after 24 h. (**c**) Cytotxicity of the bare TPM micelles toward human normal cells Detroit 551. (**d**) Fluorescent intensity of Fluorescein-labeled Doxed-loaded mixed micelles within human lung cancer cells A549. (**e**) The intracellular drug releasing behaviors of Cy 5.5-labeled Dox-loaded TPM micelles in human lung cancer A549 cells were also observed. The fluorescent dye Cy 5.5-labeled Dox-loaded TPM mixed micelles were co-cultured with A549 cancer cells. After 1 and 3 h treatment, the cells were washed with PBS. The lysosomes and cell nucleus were respectively labeled with fluorescent dye lysotracker and DAPI. The fluorescence within cancer cells after 1 and 3 h incubation was detected using a confocal laser scanning microscopy (CLSM). The Dox and Cy 5.5 fluorescences were respectively present in red and grey in the CLSM images, and the fluorescences of lysotracker and DAPI were shown in green and blue (**p < 0.01).

In this study, we exploited the toxicity of free Dox, Dox-loaded TPH and TPM micelles toward 2 different genomes of human lung cancer cells, including lung squamous cancer cell CH27 and human adenocarcinomic A549 cells. The cell viabilities of the cells after free Dox and Dox-loaded mixed micelles treatment was determined using a (3-(4,5-dimethylthiazol-2-yl)-2,5-diphenyltetrazolium bromide (MTT) assay, presented in Figure 4a,b. The cell growth was not inhibited with Dox-loaded TPH mixed micelles, whereas the cells after treatment with free Dox and Dox-loaded TPM mixed micelles revealed dose-dependent inhibition. The half-maximal inhibitory concentration (IC_50_) of free Dox toward A549 and CH27 cells was approximately 4 μg/mL. The IC_50_ values of the Dox-loaded TPM mixed micelles toward A549 and CH27 cells were 4.28 and 6.37 μg/mL, respectively. The bare polymeric mixed micelles were also treated with human normal fibroblast cells (Detroit 551 cells). The treated concentration of the bare polymeric mixed micelles was adjusted based on the drug contents, and the cell viability of the bare polymeric mixed micelles was also determined using MTT assay. The results of TPH and TPM mixed micelles, in Figure 4c and Figure S6 in the Supporting Information, point out that very low toxicity of the mixed micelles was detected. The cell death was less than 10% when the cells were treated with 1 mg/mL of the bare TPM mixed micelles. When the cells were treated with 2 mg/mL of the bare TPM mixed micelles, the cell viability was still over 85%. Detroit 551 cells exhibited 90% survival rate after being treated with 0.36 mg/mL of bare TPH mixed micelles, and when the cells were treated with lower levels of bare TPH mixed micelles, the cytotoxicity was hardly detected. In order to confirm the cytotoxicity of the Dox-loaded TPH and TPM micelles, another carcinoma was also tested. The cytotoxicity of human colon cancer cells HCT116 was detected to evaluate the doxorubicin efficacy toward cancer cells with an MTT assay, shown in Figure S7 in the Supporting Information. Dox-loaded TPH mixed micelles still exhibited very low toxicity toward cancer cells. The IC_50_ of the HCT116 cells given with free Dox and Dox-loaded TPM mixed micelles was around 10 mg/mL, higher than that of the human lung cancer cells. Cancerous cells tolerated the anticancer agent doxorubicin distinctly from other cellular types. Therefore, this result firmly indicates that the doxorubicin was physically encapsulated into TPM micelles and dominated the tumor inhibitory effects toward different cancer cellular type, instead of the TPM mixed micelles. In this study, the human lung cancer cells A549 exhibited a great response to free Dox and Dox-loaded TPM mixed micelles. Herein, the human lung cancer cells A549 were primarily tested in our advancing assessment.

The in vitro cytotoxic tests show interesting results in Dox-loaded TPH and TPM mixed micelles. Dox-loaded TPM mixed micelles exhibited tumor inhibitory effects in lung and colon cancer cell lines, while Dox-loaded TPH mixed micelles did not show any cytotoxicity, even when the same Dox concentration within the mixed micelles was given. To further investigate the cytotoxic mechanism, the endocytosis and intracellular drug-release behavior were evaluated. For internalizing the investigation into cancer cells, a fluorescent dye, 5,6-carboxyfluorescein succinimidyl ester (Fluorescein-NHS ester), was conjugated onto the TPH and TPM mixed micelles and treated with the human lung cancer cells A549, and the intracellular fluorescence was determined using flow cytometry. Prior to Fluorescein conjugation, the TPGS was first modified into TPGS-NH_2_. The terminal hydroxyl group of TPGS reacted with the carboxylic groups in cysteine via ester linkage, as Figure S8a in the Supporting Information presents. After purification, the modified TPGS-NH_2_ was characterized by a hydrogen nuclear magnetic resonance (^1^H-NMR) and Fourier transform infrared spectroscopy (FTIR), shown in Figure S8b in the Supporting Information. The conversion rate of the TPGS-NH_2_ was around 72.2%, calculating from the ethylene groups (-CH_2_CH_2_-) on the PEG segments in TPGS at 3.5–3.65 ppm and the methyl group (-CH_2_-) on the cysteine at 3–3.1 ppm. The FTIR spectrum in Figure S8c in the Supporting Information shows a peak at 1541 cm^−1^, representing the N–H bending at the modified TPGS-NH_2_ polymer [26]. The amine groups were thus successfully modified onto the TPGS for fluorescent dye labeling.

The TPGS-NH_2_ polymer was assembled with PBLG into TPH and TPM polymeric mixed micelles using the solvent exchange method, as mentioned above. The amide bonds in these mixed micelles were reacted with the NHS ester groups in Fluorescein. In order to dismiss the cell abnormality and fluorescent interference from Dox, only bare TPH and TPM mixed micelles were labeled with Fluorescein dye. After removal of the excess Fluorescein dye, the fluorescence in TPH and TPM mixed micelles was adjusted until it was the same, and the Fluorescein-labeled TPH and TPM mixed micelles were treated with human lung cancer A549 cells for 1 and 3 h. The result in Figure 4d shows that the fluorescence intensity increased with the incubation time. The fluorescence intensity increased by 10 times after the cells were treated with Fluorescein-labeled TPM mixed micelles for 1 h, whereas 19-fold fluorescence intensity was detected in cells when treated with Fluorescein-labeled TPH mixed micelles. The fluorescence intensity increased to 13-fold at 3 h post-treatment with Fluorescein-labeled TPM mixed micelles, and the increasing fluorescence intensity represented the internalization of mixed micelles into lung cancer cells. The fluorescence within A549 cells slightly increased when the cells were treated with Fluorescein-labeled TPH mixed micelles and incubated for 3 h. The overall fluorescence intensity of cells treated with TPH mixed micelles was higher than that of cells treated with TPM mixed micelles.

Since the mixed micelles have the ability to be internalized into human lung cancer A549 cells, and cytotoxicity toward cancer cells caused from TPH and TPM mixed micelles is totally different, the intracellular drug-release behaviors in our study are further discussed. The Dox intracellular releasing was observed using confocal laser scanning microscopy (CLSM). The modified polymer TPGS-NH_2_ was assembled with PBLG and the anticancer drug Dox. Later, the fluorescent dye cyanine 5.5 (Cy 5.5) was labeled onto the Dox-loaded mixed micelles following the previous methods. Before treatment, the Cy 5.5-labeled Dox-loaded TPH and TPM mixed micelles were adjusted based on the Dox concentration. Thereafter, Cy 5.5-labeled Dox-loaded mixed micelles were treated with A549 cells for 1 and 3 h. The cells were further stained with fluorescent dye, including lysotracker Red DND-99 and 4′,6-diamidino-2-phenylindole (DAPI), to respectively symbolize the cellular lysosomes and nuclei. The fluorescence of Cy 5.5 dye and the anticancer drug doxorubicin is presented in grey and red colors, respectively, in Figure 4e, whereas the fluorescence for determining lysosome and cellar nuclei is presented in green and blue colors, respectively. Figure 4e shows the Cy 5.5 fluorescence overlapping with the lysosome fluorescence after 1 h incubation, in agreement with the internalization of Dox-loaded TPH and TPM mixed micelles. The higher fluorescence intensity of Cy 5.5 fluorescence was observed in cells treated with Cy 5.5-labeled Dox-loaded mixed micelles because the higher levels of modified TPGS-NH_2_, which enabled conjugation with fluorescent dye Cy 5.5 and the TPM micelles. After Cy 5.5-labeled Dox-loaded TPM mixed micelles were co-cultured with cells for 3 h, the red colors can be significantly observed as overlapping with the fluorescence of Cy 5.5 (grey) and lysosomes (green). This demonstrates that mixed micelles moved toward lysosomes and the Dox was released. The Dox-loaded TPM mixed micelles were able to release their payloads at the acidic environment (pH 4.0), as the acidity of the lysosome may lead to the secondary conversion, intracellularly liberating the Dox. However, the intracellular drug-releasing behavior was not witnessed in cells incubated with Cy 5.5-labeled Dox-loaded TPH mixed micelles for 3 h. Weak Dox fluorescence (red) overlaps with the fluorescence of the lysosome (green), indicating only little Dox was released from TPH mixed micelles, even though the Cy 5.5 fluorescence on TPH mixed micelles increased upon incubation time. The CLSM images could clearly interpret the different intracellular drug-releasing behaviors between the TPH and TPM mixed micelles that primarily affect the cytotoxicity of Dox-loaded mixed micelles toward cancer cells. The severe cytotoxicity of Dox-loaded TPM mixed micelles determined from MTT assay could be recognized as the consequence of the internalization and intracellular drug-releasing behaviors of the Dox-loaded mixed micelles. Eventually, Dox-loaded TPM mixed micelles, which have ability to inhibit cancer cell growth by intracellular drug releasing, were selected to further perform the in vivo tests for anticancer feasibility.

### 2.5. Tumor Accumulation and In Vivo Antitumor Efficacy

**Figure 5:**
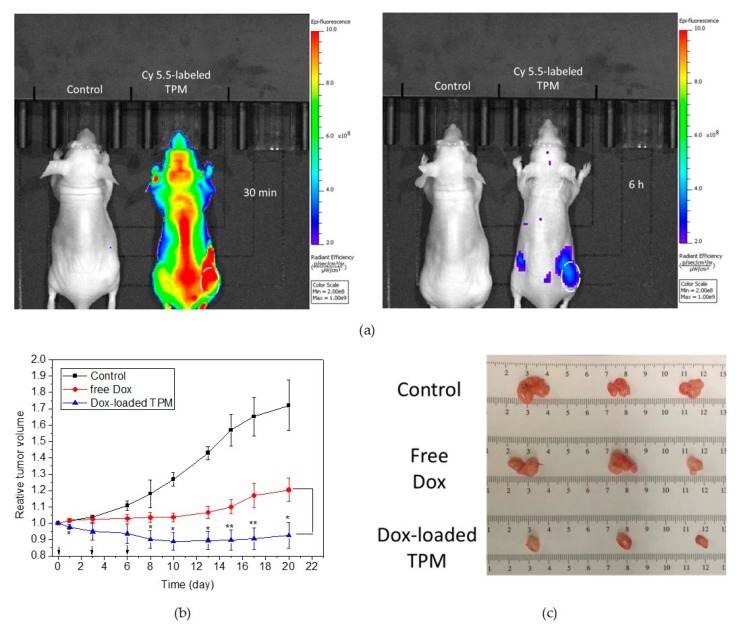
In vivo evaluation of the secondary structure contained mixed micelles. (**a**) The optical florescence of human lung cancer A549 bearing nude mice at post 30 min and 6 h injection with Cy5.5-labeled TPM micelles. The fluorescent intensity of the tumor marked. (**b**) Antitumor efficacy in A549 cells-inoculating nude mice. The tumor-bearing nude mice were divided into 3 groups and independently intravenously administered with PBS, doxorubicin and Dox-loaded mixed micelles at a 10 mg/kg/mL of Dox equivalent dosage each time. Total three dosages were given, present in arrows. The tumor sizes were monitored and the comparison with each group was statically analysis. The asterisk markers represented the statically significant differences (*p < 0.05 and **p < 0.01). (**c**) After 20 d treatment, the mice were sacrifice and the tumor tissues were collected and photographed.

The tumor accumulating behaviors were surveyed in this study by A549 cells-bearing nude mice models. Human lung cancer A549 cells were xenografted onto the back of the 4 week old female nude mice. Mice were provided by National Laboratory Animal Center (NLAC), NARLabs, Taiwan. When the tumor volume reached 50–100 mm^3^, the mice were intravenously injected with Cy 5.5-labled TPM mixed micelles. At 30 min and 6 h post-injection, the Cy 5.5 fluorescence in mice was observed using IVIS, shown in Figure 5a. The Cy 5.5 fluorescence was observed spreading over the A549 bearing nude mice after 30 min injection. At 6 h post-administration, the Cy 5.5 fluorescence was mainly deposited in the tumor sites, showing that the TPM micelles were stably prone to accumulating in the tumors.

The A549 cells-bearing nude mice models were also employed to realize the tumor inhibition of Dox-loaded TPM mixed micelles in vivo. When the tumor volume grew to 50 mm^3^, the tumor-inoculated mice were separated into 3 groups, with 3 mice per group. The mice in these 3 groups were independently intravenously injected with PBS (named control), free Dox (10 mg/kg) and Dox-loaded TPM micelles (10 mg/kg, adjusted on the Dox concentration) at days 0, 3, and 6. The tumor sizes of these mice were measured and recorded, as presented in Figure 5b. The tumor sizes of the mice in the control group increased over time. Twenty days later, the average tumor sizes grew two times bigger than those at day 0. The tumor growth in those mice treated with free Dox was retarded at first. At day 3 and 6, the tumor sizes approached those in mice administered with Dox-loaded TPM micelles. However, the rates of tumor growth increased when free Dox was not affordable to those mice and, after day 8, the tumor sizes were significantly larger than those in mice treated with Dox-loaded TPM micelles. At 20 d post-first injection, the tumor sizes enlarged 1.2-fold. The tumor growths of those mice applied with Dox-loaded TPM mixed micelles were inhibited during these 20 days. Before day 10, the tumor sizes dramatically reduced due to the continuous Dox-loaded micelles administration. Thereafter, the tumor sizes slightly increased, but the tumors grew slowly. The tumors were collected and photographed after the mice were sacrificed at day 20, in Figure 5c. The tumors from the mice treated with Dox-loaded TPM micelles were the smallest among all the specimens. That could be explained by the efficient tumor deposit of the Dox-loaded TPM micelles and Dox intracellularly release.

The body weights of all the mice were also monitored and the results did not exhibit the statistical significance. However, in order to further evaluate the biosafety, blood samples were also collected at day 20 and the biochemical indexes were examined, in particular for renal and hepatic functions. The hepatic functions could be evaluated through glutamic–oxalocetic transaminase (GOT) and glutamic–pyruvic transaminase (GPT) indexes, whereas the renal functions could be assessed with blood urea nitrogen (BUN) and creatinine values. The examination results are shown in Table 2. The normal references for GOT and GPT in mice were 28–132 U/L and 59–247 U/L, respectively [27]. The GOT values in mice with a PBS injection (control group) were 216 ± 43 U/L on average, whereas the GOT average values in mice applied with free Dox were 225 ± 50 U/L and those in mice with Dox-loaded TPM miceller treatment were 187 ± 43 U/L. The GOT values were all higher than the normal indexes. The GPT values for the mice in the control, free Dox and Dox-loaded TPM micelles groups were 91 ± 21, 95 ± 11, and 90 ± 26 U/L, respectively. The BUN values of all the mice in the control, free Dox and Dox-loaded TPM micelles groups were approximately 31 mg/dL on average. Nevertheless, the normal value of the BUN ranged from 17 to 28 mg/dL [27] and the BUN of all mice was a little higher than the normal range. Another biochemical index creatinine was utilized to evaluate the renal function. The creatinine values in all mice were around 0.4 mg/dL, while the normal creatinine value is 0.2–0.8 mg/dL [27]. According to the renal and hepatic functional indexes and the body weight, Dox-loaded TPM micelles did not cause severe damage to the kidney and liver, which are considered to be affected by nanoparticles [28,29].

## 3. Discussion

In this study, the mixed micelles containing secondary structures were prepared. The secondary conformation of PBLG has been reported as being affected by blended polymers via hydrogen or π–π stacking interactions [10,11]. In a mixed micellar system, R. Mondal et al. further identified amphiphilic copolymers to have the ability to influence the secondary structures [30]. J. Atkinson illustrated that amphiphiles assist the secondary folding [31]. S. Kuo et al. also indicated that phenolic polymers induce the intramolecular hydrogen interaction formation and affected the helix ratios when blending with PBLG polymers in solid state [10]. In our study, the mixed micelles were identified by the DSC analysis, as shown in the Supporting Information, identifying the participation of the amphiphilic TPGS and hydrophobic PBLG in the micelles. The CD spectrum in Figure 1b showed the helix-coil characteristic peaks of all mixed micelles, representing that PBLG still could fold into secondary structures in existence with amphiphilic TPGS in a mixed micellar system. The magnitude of the 222 nm valley negatively increased upon the introduction of the PBLG to the mixed micellar system, indicating that the helix structures dominantly came from PBLG and the insertion of TPGS may interfere the helical arrangement in our mixed micellar system.

Generally, the lower CMC value may increase the stability of the micelles. In this study, the highest contents of the PBLG in mixed micelles exhibited the lowest CMC value and better stability (TPH mixed micelles), because of the highest ratio of the helical conformation, in consistency with the results from J. Ding et al. [32]. However, TPM mixed micelles, having the lowest CMC value, rather exhibited high stability, due to the α-helix conformation. The ordered secondary structures may facilitate the crystalline alignment, as S. Funari et al. reported, due to the reduction of the elastic properties in polymers [33], whereas the crystalline microphase of the hydrophobic segments plays a determinant role in micellar stability [34]. The concentrated PBLG, as comprising in a micellar system, induced the crystalline alignment [35]. Therefore, it is not surprising to discover the crystalline formations by XRD analysis within a mixed micellar system, in Figure 2c,f [35]. In our case, the crystalline structure would align in a lamellar manner and grow out-of-plane [24]. WARD results show that 2 θ = 2 and 6, showing that the distances between the α-helices of the PBLG and the pitch length of α-helices were 4.415 and 1.472 nm, respectively. The latter value was close to that reported by S. Kuo et al., identifying the α-helix conformation of PBLG in the mixed micelles; the former was much larger than that reported by S. Kuo et al., resulting from the TPGS random coil insertion into the mixed micelles [35]. Although the crystallinity and secondary structures were embedded inside the mixed micelles, based on pevious studies from S. Funari et al., the crystalline core of the mixed micelles still could respond to outer temperature stimuli [10]. Y. Mochida et al. also announced that the helix core bundled inside the micelles could still release the payload in response to the external conditions [14]. The reducing or disappearing secondary structures and core crystalline as R. Sallach et al. indicated, led to a loosening of the density of the micellar core and the releasing of the payloads [15]. In Figure 1b, the mixed micelles could be discovered their rearrangement of inner structures in response to outer temperature by the alternation in magnitude of secondary structure. Further, in Figure 2b,c, the inner secondary structures as well as crystallinity responded to the pH values of outer environment. For PBLG, the secondary structures or crystalline alignments were reported being influenced by the protonation toward the environments, though the mechanism is not completely clear [8,9]. Nevertheless, the pH sensitivity of PBLG was mostly neglected or seldom discussed in other polymeric micellar systems [1,23,25]. This might be because the terminus of PBLG was modified or conjugated onto another hydrophilic polymer in most drug or gene delivery systems. T. Itoh et al. indicated that the terminus of PBLG and the neighboring carboxylic or amine regions lack intrahelical hydrogen bonds, leading to the helix coil interconversion in response to the environment [8]. In our study, the terminals were embedded within the micellar structure, hence exerting significantly pH-responsive properties in the mixed micelles.

Small molecules of doxorubicin were encapsulated in the mixed micelles. The effect of the small molecules on the secondary structures was inconclusive [36,37]. Small amount of Dox led to the negatively increments in magnitude at 222 nm in the CD spectrum, showing the helix contents increased after Dox loading into the mixed micelles, in comparison of the bare mixed micellar carrier. That could be attributed to the π–π stacking interactions between the doxorubicin and the hydrophobic core of the mixed micelles. However, the CD spectrum in Figure 3c,d also indicates that high levels of Dox encapsulation led to the reduction of helical alignment within the mixed micelles. In our study, only a little Dox leaked from the mixed micelles at pH 7.4, according to the drug releasing profiles, as shown in Figure 3e, while in most mixed micellar system, particularly composed of TPGS, burst release occurred within the first 4 h [38,39]. Less drug leakage of our mixed micellar system in neutral conditions could be attributed to the crystalline core [34,40,41], aligned from the PBLG’s secondary conformation. In a pH 4.0 environment, the secondary structures in Dox-loaded TPM mixed micelles, as well as the crystalline alignments, were driven into disorder. Dox-loaded TPH mixed micelles, which possessed highly crystalline alignment at the core, however, did less response to the environmental pH values, because their crystallinity could not be regulated by the environmental pH, probably. The crystalline microphase inside a drug carrier, as J. Jeong et al. reported, would reduce the Dox diffusion out of the drug carriers and hence retard the drug release [42]. Consequently, the drug could be released from the semicrystalline mixed micelles (TPM mixed micelles) in pH 4.0 faster than in pH 7.4.

Doxorubicin is currently applied in lung and colon cancers in clinic [43,44,45]. In our study, the cytotoxicities of the Dox-loaded mixed micelles toward human lung cancer cell lines A549, CH27, and human colon cancer cell line HCT116 were independently evaluated by MTT assay. Among all the cells tests, A549 cells superiorly sensitize with Dox-loaded TPM mixed micelles, while the IC_50_ values of the both human lung cancer cell lines were similar, as shown in Figure 4b. The reason for this might be the rapid internalization or the fast-intracellular drug release. Human colon cancer HCT116 cells, which are considered to be less response to doxorubicin [45], exhibited the lowest cell death among the three cell lines (in the Supporting Information). The results prove that the doxorubicin was physically encapsulated into mixed micelles. Considering the future application, we focus on the A549 cell line, which was the most sensitive cells among these three cell lines to deeply investigate. Notably, Dox-loaded TPH mixed micelles were also treated with these cell lines, while almost no cytotoxicity was shown and the reasons were further studied. The internalization of the mixed micelles into A549 cells was investigated. Numerous studies have revealed that a well-defined conformation peptide chain exhibited a cell penetrating ability [46,47,48]. The mixed micelles in this study could be detected as rapidly entering into cancer cells by flow cytometry, as Figure 4d shows. TPH mixed micelles, having high contents of the helical structures, showed faster uptake into A549 cells than TPM micelles. M. Oba et al. further identified that the secondary conformation is altered during endocytosis [49], demonstrating that the inner secondary structures within the mixed micelles are altered with the pH values during internalization. The CLSM images in Figure 4e show that the doxorubicin is liberated from TPM mixed micelles during endocytosis, due to the disordered secondary folding and the disruption of the crystalline alignment in response to pH values. However, Figure 4e indicates that little Dox could be released from TPH mixed micelles in the process of internalization. This could account for their lack of secondary structure regulation and crystallinity. The in vitro results pictured show that the Dox-loaded mixed micelles could be taken up by cancer cells and the payloads would be intracellularly released.

The tumor accumulating behaviors of the mixed micelles were also investigated. The mixed micelles were able to rapidly accumulate in tumor tissue, while at 6 h post-administration, most mixed micelles were observed as being eliminated from mice. Y. Noguchi demonstrated that N-(2-hydroxypropyl) methacrylamide (HPMA) macromolecules also revealed a rapid tumor deposit within 10 min. After 6 h, some of them, in particular lower molecular weights of the macromolecules, would be totally perfused into the blood and clearance [50], while the higher molecular weight copolymers, which were later proved as having better tumor anti-proliferation [51], accumulate mainly at the tumor site. Our secondary conformation containing mixed micelles which were mainly observed as being deposited in the tumor tissues after 6 h treatment, illustrating their potential in antitumor treatment. The in vivo antitumor inhibition identified the efficient antitumor effects. After treatment, although the hepatic index GOT and the renal index BUN of all mice were higher than the normal references, there was, notably, no significant difference between the groups, showing that the abnormalities were not launched from the Dox treatment. In our system, the crystalline core efficiently prohibited the burst release [38,39], retaining more active drugs being transported into the tumors. As the mixed micelles containing the secondary structures have shown exceptional antineoplastic efficacy in vitro and in vivo with low toxicity, the novel micelles would be worth being further developed.

## 4. Materials and Methods

### 4.1. Materials

Poly-γ-benzyl-l-glutamate (PBLG) (M.W. 30,000–70,000), d-α-tocopherol polyethylene glycol 1000 succinate (TPGS), triethylamine (TEA), 1-ethyl-3-(3-dimethylaminopropyl) carbodiimide (EDC) and MTT reagents were obtained from Sigma-Aldrich Co., LTD (St. Louis, MO, USA). ACS-grade organic solvents including N, N-dimethylacetamide (DMAc) and tetrahydrofuran (THF) were respectively purchased from Duksan pure chemicals (Ansan City, South Korea) and Merck (Darmstadt, Germany). Chemical reagent 4-dimethylaminopyridine (DMAP) was purchased from Alfa Aesar (Lancashire, UK) and doxorubicin hydrochloride (Dox-HCl) was purchased from Tokyo Chemical Industry Co., LTD (Tokyo, Japan). Phosphotungstic acid (PTA) for TEM staining was prepared from sodium phosphotungstate hydrate, which was acquired from Acros Organics (Geel, Belgium). Fluorescent dye lysotracker Red DND-99 and 5,6-carboxyfluorescein succinimidyl ester (Fluorescein-NHS ester) were both purchased from Thermo Fisher Co., Ltd. (Waltham, MA, USA). The other fluorescent dye cyanine 5.5 NHS ester (Cy 5.5-NHS ester) was obtained from Lumiprobe (Hunt Valley, MD, USA). Fluorescence 4′,6-diamidino-2-phenylindole contained mounting medium (DAPI-containing mounting medium) was purchased from Cisbio (Parc Marcel Boiteux, France). The animal tests were approved by Institutional Animal Care and Use Committee (IACUC approval number: CMUIACUC-2018-154-1) in China Medical University. The materials in our animal tests, including Matrigel and isoflurane were respectively obtained from Merck (Darmstadt, Germany) and Panion and BF Biotech. Inc. (Taiwan).

### 4.2. Preparation and Characterizations of TPGS/PBLG Polymeric Mixed Micelles

Polymers including PBLG and TPGS were weighed at various ratios and dissolved with DMAc (8 mL totally). The solutions were placed into the dialysis bags (M.W. C.O. 1k) and dialyzed against deionized water at room temperature with changing water for 6 times, forming TPGS/PBLG polymeric mixed micelles. After dialysis, the particle sizes of TPGS/PBLG polymeric mixed micelles were determined using dynamic laser scattering (DLS) (Malvern ZS90).

Hydrophobic fluorescent probe pyrene was dissolved into acetone (6 × 10^−5^ M) and diluted with deionized water until it reached 1.2 × 10^−6^ M. The organic solvent acetone was evaporated using a rotary evaporation machine. Simultaneously, polymers including PBLG and TPGS were, dissolved into THF and prepared into 2 mg/mL of polymer solutions. The solution was slowly added into the deionized water and the organic solvent THF was eliminated using a rotary evaporation machine. The PBLG and TPGS solution were thereafter diluted and mixed together under the ratios of micellar components. The polymers were serially diluted from 0.5 to 9.6 × 10^−4^ mg/mL. The diluted polymer solution was then taken and mixed with the equal volume of the pyrene solution. After being stored in a dark place for 1 d, the fluorescence intensities of these solutions were measured with a fluorescence reader (SpectraMax iD3). The wavelengths at 337 nm (I_337_) and 335 nm (I_335_) were utilized for excitation and the fluorescence wavelength at 393 nm was detected.

The secondary structures of the TPGS/PBLG polymeric mixed micelles were characterized by the circular dichroism spectrum (CD spectrum) (JASCO J-815 spectropolarimeter). The crystalline alignment was characterized using a high-resolution x-ray diffractometer (HRXRD) (Bruker D8 SSS) and assessed by a differential scanning calorimetry (DSC) (Netzsch 200F3) from 30 °C to 350 °C with the nitrogen flow.

### 4.3. Stability and pH Responsive Behaviors

The polymeric mixed micelles were independently incubated at 37 °C in pH 7.4 and pH 4.0 conditions. At 6 h and 24 h later, the hydrodynamic diameters of the polymeric mixed micelles were measured by DLS. The chemical or physical interactions within micelles were also determined by gel permeation chromatography (GPC) after incubation at different pH value after 24 h. The secondary structures after 6 h and 24 h incubation were simultaneously predicted using the CD spectrum. The crystalline alignment was determined by HRXRD and DSC analysis. The polymeric mixed micelles were incubated with pH 7.4 and pH 4.0 conditions, respectively. After 24 h, the polymeric mixed micelles were dropped onto the copper grid and stained by 1 wt% of phosphotungstic acid (PTA). After the removal of the excess sample and staining reagents, the copper grids were dried and stored at room temperature. The morphologies of the polymeric mixed micelles were observed using transmission electron microscopy (TEM) (JEOL JEM-1400) with the accelerated voltages. The samples were also frozen with liquid methane and observed using cryo-electron microscopy (CryoEM) (FEI Tecnai G2 F20 TWIN). 

### 4.4. Drug Loading and Releasing Behavior Study

The anticancer drug doxorubicin (1 mmole) was dissolved into DMAc and TEA was added into the solutions. The solutions were stirred and reacted for 2 h. After 2 h, the anticancer drug doxorubicin was weighed and dissolved into DMAc with the TPGS and PBLG. The solutions were thereafter placed into the dialysis bags (M.W. C.O. 1k) and dialyzed against deionized water. After dialysis, the Dox-loaded polymeric mixed micelles were collected and stored at 4 °C.

Dox-loaded mixed micelles were placed into an ultracentrifugal tube (M.W.C.O 10 k) (Pall Corporation, Port Washington, NY, USA) and their pH values were adjusted to 7.4 or 4.0 using 0.1 M of HCl aqueous solution. The ultracentrifugal tube was placed at 37 °C under shaking. At 1, 2, 4, 6, 12 and 24 h post-incubation, the releasing doxorubicin was collected after centrifugation. The concentration of doxorubicin was detected using an ELISA reader (Biotek Synergy HT) at 488 nm wavelength. The secondary conformation of the Dox-loaded mixed micelles was also monitored using CD spectroscopy.

### 4.5. In Vitro Cytotoxicity Assessment

The cytotoxicity toward human cancer cells was assessed using MTT assay. Human squamous lung cancer CH27, epithelial lung cancer A549 and colon cancer HCT116 cells (1 × 10^5^ cells/mL) were seeded each well in a 96-well plate and incubated at 37 °C with 5% CO_2_ supply. As the cells attached, the cells were treated with predetermined concentrations from 10.00 to 0.63 μg/mL of free Dox and Dox-loaded mixed micelles with sequential dilutions 0.63, 1.25, 2.5, 5.00, and 10.00 μg/mL. After being co-cultured for 24 h, the excess free Dox solution and Dox-loaded mixed micelles were removed and the cells were washed with PBS twice. The MTT reagent was applied and the enzyme-linked immunosorbent assay (ELISA) reader (Biotek Synergy HT) was utilized for cell viability determination.

In addition, the cytotoxicity of the bare polymeric mixed micelles was also determined by MTT assay. Human normal fibroblast cells Detroit 551 were (1 × 10^5^ cells/mL) seeded each well in a 96-well plate and placed at 37 °C with 5% CO_2_ supply. When the cells attached onto the plate, the bare TPM polymeric mixed micelles (0.13, 0.25, 0.5, 1.00, and 2.00 mg/mL) and TPH (0.02, 0.05, 0.09, 0.18, and 0.36 mg/mL) micelles were treated with Detroit 551 cells and the cells were incubated at 37 °C with 5% CO_2_ supply. Twenty-four hours later, the bare polymeric mixed micelles were eliminated and cells were washed with PBS twice. The MTT assay was also utilized for cytotoxic assessment.

### 4.6. Internalization and Intracellular Drug Releasing Observation

In order to label the fluorescence, TPGS was modified into amine groups after reacting with the cysteine via ester bond conjugation. The modification of TPGS was performed by the following method: TPGS (1 mmole) and cysteine (1 mmole) were weighed and dissolved with deionized water in sample vials. DMAP (0.1 mmole) and EDC (0.3 mmole) were also dissolved into deionized water and slowly added into the TPGS and cysteine mixtures. After stirring at 25 °C for 24 h, the TPGS solution was passed through the PD-10 desalting column for purification. The products were characterized by ^1^H-NMR and FT-IR.

The prepared TPGS-NH_2_ and PBLG were dissolved into the DMAc. The solutions were prepared for polymeric mixed micelles using solvent exchange methods, as mentioned above. The polymeric mixed micelle solutions were collected and mixed with the fluorescent dye Fluorescein-NHS ester at 25 °C for 24 h. Afterwards, the micellar solution was placed into a dialysis bags (M.W.C.O 6–8 k) and dialyzed against deionized water for purification. The Fluorescein-labeled polymeric mixed micelles were collected and stored at 4 °C.

Human lung cancer cells A549 (1 × 10^6^ cells/mL) were seeded onto the 6-well plate. As the cells attached onto the plate, the Fluorescein-loaded polymeric mixed micelles were treated with the A549 cells at 37 °C with 5% CO_2_ supply. After 1 and 3 h incubation [52], the excess Fluorescein-loaded polymeric mixed micelles were removed and the cells were washed with phosphate buffering saline (PBS) twice. The cells were thereafter collected using centrifugation. The harvested cells were placed into testing tubes on ice. Thereafter, 1 × 10^4^ cells were randomly chosen and the Fluorescein intensity was determined using flow cytometry (BD FACS Canto, East Rutherford, NJ, USA) [53] to evaluate the internalization of the mixed micelles.

TPGS-NH_2_ was further assembled into polymeric mixed micelles with PBLG and doxorubicin, following the methods described above. After the dialysis, the polymeric mixed micelles were mixed with the fluorescent dye Cy 5.5-NHS ester at 37 °C with stirring for 24 h. The excess Cy 5.5-NHS ester was eliminated by dialysis. Human lung cancer cells A549 were seeded onto a 6-well plate. As the cells attached, the Cy 5.5-labeled Dox-loaded polymeric mixed micelles were incubated with the cells at 37 °C with 5% CO_2_ supply. At 1 and 3 h post-treatment, the Cy 5.5-labeled Dox-loaded polymeric mixed micelles were removed and the cells were washed with PBS thrice. The fluorescent dye lysotracker Red DND-99 (1 μM) was treated with A549 cells for 1 h at 37 °C with 5% CO_2_ supply. After treatment, the excess lysotracker fluorescent dye was removed. After washing the cells using PBS thrice, the cells were fixed with 4% of paraformaldehyde. Twenty min later, the cells were washed with PBS and the cells were mounted with DAPI-containing mounting medium. The cells were observed using confocal laser scanning microscopy (CLSM) (Leica TCS SP 8, Germany). The fluorescences of Cy 5.5 and lysotracker Red DND-99 were detected using the excitation wavelength at 633 nm and 570 nm and the emission wavelength at 650 nm and 590 nm, respectively. The fluorescence of doxorubicin was observed at the excitation wavelength of 488 nm and the emission wavelength of 520 nm. DAPI was observed using the approximate excitation and emission wavelength, preset in the equipment.

### 4.7. Tumor Deposit and In Vivo Antitumor Efficacy

The xenografted human lung cancer A549 cells nude mice model was established to assess the tumor accumulating behaviors and the in vivo antitumor efficacy. Human lung cancer A549 cells (1 × 10^7^ cells/mL) were homogenously mixed with an equal volume of Matrigel solution and the mixture (0.1 mL) was subcutaneously transplanted into the back of each mouse. As the tumor volume achieved 500 mm^3^ or above, the tumor-inoculated nude mice were intravenously administered with Cy 5.5-labeled TPM mixed micelles. At 30 min and 6 h post-injection, the mice were anesthetized with 1.5% of isoflurane and the optical fluorescence was observed using an in vivo imaging system (IVIS) (Lumina LT system) with an excitation length at 640 nm and a Cy 5.5 filter channel.

The A549 tumor-bearing mice model was also exploited in antitumor assessment, following the abovementioned methods. When the tumor volume grew to 500 mm^3^, the mice were randomly divided into 3 groups, with 3 mice per group. The mice in each group were independently injected with PBS, 10 mg/kg of free Dox and 10 mg/kg of Dox-loaded TPM micelles (the concentration was previously adjusted according to the Dox concentration) via their tail veins at day 0, 2, and 6. The tumor sizes and weights were monitored every 2 or 3 days. The tumor sizes were measured using a caliper and calculated as the following formula: Tumor size (mm^3^) = ab^2^/2, where a and b, respectively, represent the length and width of the tumor. Twenty days later, all of the mice were sacrificed, and CO_2_ and blood samples were taken. The blood was centrifuged under 3000 r.p.m. for 10 min and the supernatant serum was obtained to analyze the hepatic and renal functions by an automatic analyzer (Hitachi-7150, Japan) with the commercial kits provided by DiaSys Diagnostic Systems GmbH (Germany). The glutamic–oxalocetic transaminase (GOT) and glutamic–pyruvic transaminase (GPT) indexes were utilized to evaluate the hepatic functions, whereas the renal functions were presented by the blood urea nitrogen (BUN) and creatinine (CRE) indexes, according to the manufacturer’s instructions.

### 4.8. Statistical Analysis

All results are shown in average values and standard deviation, presented in mean ± S.D. (standard deviation). All data were statistically analyzed using Student’s t-test (Microsoft Excel 2000). The significant differences were considered when the *p*-value was less than 0.05 (*p* < 0.05). The significant differences are shown in star marks (*p < 0.05; **p < 0.01 and ***p < 0.001).

## 5. Conclusions

For the first time, we revealed and fabricated a pH-tunable secondary structure containing mixed micelles (TPGS and PBLG) for controlled intracellular anticancer drug delivery. Importantly, the drug loading and releasing using these micelles could be precisely controlled through the regulation of the pH value, via the embedded PBLG. In the neutral condition, α-helix conformations of PBLG formed the crystalline structures to stabilize the mixed micelles with the loaded anticancer drug Dox; meanwhile, in acidic milieus (pH 4.0), the secondary conformation would transform into a random-coil state and disorder the inner crystalline alignment in the mixed micelles, allowing the drug release. The Dox-loaded pH-tunable mixed micelles would be internalized into cells, causing intracellular drug release and leading to cell death. The mixed micelles were also deposited in the primary tumor and the antitumor efficacy of the Dox-loaded mixed micelles was identified in vivo. The novel pH-tunable secondary conformation containing mixed micelles are, therefore, valuable for future clinical study.

## Figures and Tables

**Table 1:** The particle sizes and distributions of the mixed micelles with various compositions.

Code	Composition (wt%)		Size (nm)	PDI	CMC (mg/mL)
	TPGS	PBLG			
TPH	25	75	148.73 ± 1.27	0.09 ± 0.03	5.68 × 10^−4^
TPM	50	50	157.03 ± 3.00	0.09 ± 0.02	3.22 × 10^−3^
TPL	75	25	184.00 ± 0.72	0.07 ± 0.04	1.29 × 10^−3^

Note: The particle sizes and PDI (polydispersity) were determined by the dynamic laser scattering (DLS) and CMC (critical micellar concentration) values were determined by pyrene probe methods.

**Table 2:** Hepatic and renal function evaluation.

Code	Hepatic Function		Renal Function	
	GOT (U/L)	GPT (U/L)	BUN (mg/dL)	Creatinine (mg/dL)
Control	216 ± 43	91 ± 21	32 ± 3	0.42 ± 0.03
Free Dox	226 ± 50	95 ± 11	31 ± 3	0.39 ± 0.06
Dox-loaded TPM micelle	187 ± 42	90 ± 26	31 ± 2	0.36 ± 0.05

## Supplementary Materials

The following are available online at https://www.mdpi.com/2072-6694/12/2/503/s1, Figure S1: DSC analysis of TPGS, PBLG, TPH and TPM, Figure S2: TEM images of TPH (a) and TPM (b) mixed micelles after incubation at 37 °C for 24 h, Figure S3: The CD spectrum of TPM mixed micelles at pH 7.4, 6.5 and 4.0 conditions after incubation at 37 °C for 24h, Figure S4: DSC analysis of TPGS, PBLG, TPH and TPM at pH4.0, Figure S5: Gel permeation chromatography (GPC) of TPM at different pH values (7.4 and 4.0) after incubation at 37 °C for 6 and 24 h, Figure S6: Cell viability of bare TPH mixed micelles in human normal cells Detroit 551, Figure S7: The cytotoxicity assay of free Dox, Dox-loaded TPH and TPM micelles was independently conducted on human colon carcinoma cell lines HCT 116, Figure S8: Characterization of TPGS-NH_2_.

## Abbreviations

PBLG	poly-γ-benzyl-l-glutamate
TPGS	d-α-tocopherol polyethylene glycol succinate
Dox	doxorubicin
DMAc	dimethylacetamide
DLS	dynamic laser scattering
CD spectrum	circular dichroism spectrum
DSC	differential scanning calorimetry
T_m_	melting point
XRD	x-ray diffractometer
TEM	transmission electron microscopy
PTA	phosphotungstic acid
GPC	gel permeation chromatogram
UV–vis spectrometer	ultraviolet–visible light spectrometer
Fluorescein	5,6-carboxyfluorescein succinimidyl ester
CLSM	confocal laser scanning microscopy
